# Supplementary material for: Nodes with high centrality in protein interaction networks are responsible for driving signaling pathways in diabetic nephropathy
Source: PeerJ. 2015 Oct 1;3:e1284. doi: 10.7717/peerj.1284 (PMC4636410; doi:10.7717/peerj.1284)
Supplement: Table S1 — Forty-nine genes were differently expressed between normal and DN samples with adjusted p-value <0.05. The genes are sorted by log2 of fold change (LogFC). [file peerj-03-1284-s001.doc]

**Supplementary Table 1- Differently Expressed Genes. Forty nine genes were differently expressed between normal and DN samples with adjusted p-value< 0.05. The genes are sorted by log_2_ of fold change (LogFC).**

| **Down-Regulated Genes** | | |  | **Up-Regulated Genes** | | |
| --- | --- | --- | --- | --- | --- | --- |
| **Gene** | **Adj.P-value** | **LogFC** |  | **Gene** | **Adj.P-value** | **LogFC** |
| **CLIC5** | 0.041 | -4.381 |  | **SLC9A7** | 0.041 | 4.331 |
| **VEGFA** | 0.041 | -4.262 |  | **MPP6** | 0.050 | 4.108 |
| **HSPA12A** | 0.041 | -4.194 |  | **CEP152** | 0.041 | 4.004 |
| **NEBL** | 0.046 | -3.992 |  | **TUBB4A** | 0.046 | 3.909 |
| **PLCE1** | 0.041 | -3.980 |  | **ITGB1** | 0.044 | 3.774 |
| **SEMA5A** | 0.041 | -3.957 |  | **EGR2** | 0.044 | 3.580 |
| **PTPRO** | 0.041 | -3.922 |  | **CYP2C8** | 0.041 | 3.557 |
| **GMDS** | 0.041 | -3.900 |  | **XPNPEP2** | 0.046 | 3.455 |
| **MME** | 0.041 | -3.838 |  | **FUT6** | 0.046 | 3.357 |
| **HOXD1** | 0.041 | -3.813 |  | **RFC4** | 0.048 | 3.094 |
| **DPYSL3** | 0.046 | -3.782 |  | **CHRNE** | 0.046 | 2.954 |
| **FGF1** | 0.041 | -3.743 |  | **LCN1** | 0.046 | 2.654 |
| **GAS1** | 0.044 | -3.659 |  | **UGT2B17** | 0.046 | 2.605 |
| **AIF1** | 0.041 | -3.616 |  | **ADORA2B** | 0.043 | 2.582 |
| **TNNC1** | 0.041 | -3.602 |  | **POU2F2** | 0.046 | 2.520 |
| **FOXC1** | 0.041 | -3.506 |  | **HYAL1** | 0.046 | 2.218 |
| **PLA2R1** | 0.044 | -3.485 |  |  |  |  |
| **THBS1** | 0.041 | -3.170 |  |  |  |  |
| **BMP2** | 0.041 | -3.115 |  |  |  |  |
| **PTPRD** | 0.046 | -3.052 |  |  |  |  |
| **F3** | 0.044 | -3.036 |  |  |  |  |
| **FRY** | 0.046 | -2.922 |  |  |  |  |
| **TYRO3** | 0.044 | -2.864 |  |  |  |  |
| **ST3GAL6** | 0.041 | -2.858 |  |  |  |  |
| **F2R** | 0.041 | -2.809 |  |  |  |  |
| **CDS1** | 0.044 | -2.695 |  |  |  |  |
| **CRIM1** | 0.044 | -2.665 |  |  |  |  |
| **C1orf21** | 0.046 | -2.656 |  |  |  |  |
| **DPP6** | 0.050 | -2.641 |  |  |  |  |
| **IQGAP2** | 0.050 | -2.592 |  |  |  |  |
| **THSD7A** | 0.041 | -2.581 |  |  |  |  |
| **MGAT5** | 0.046 | -2.569 |  |  |  |  |
| **PAM** | 0.046 | -2.256 |  |  |  |  |
