# Supplementary figures and images for: Nodes with high centrality in protein interaction networks are responsible for driving signaling pathways in diabetic nephropathy
Source: PeerJ. 2015 Oct 1;3:e1284. doi: 10.7717/peerj.1284 (PMC4636410; doi:10.7717/peerj.1284)

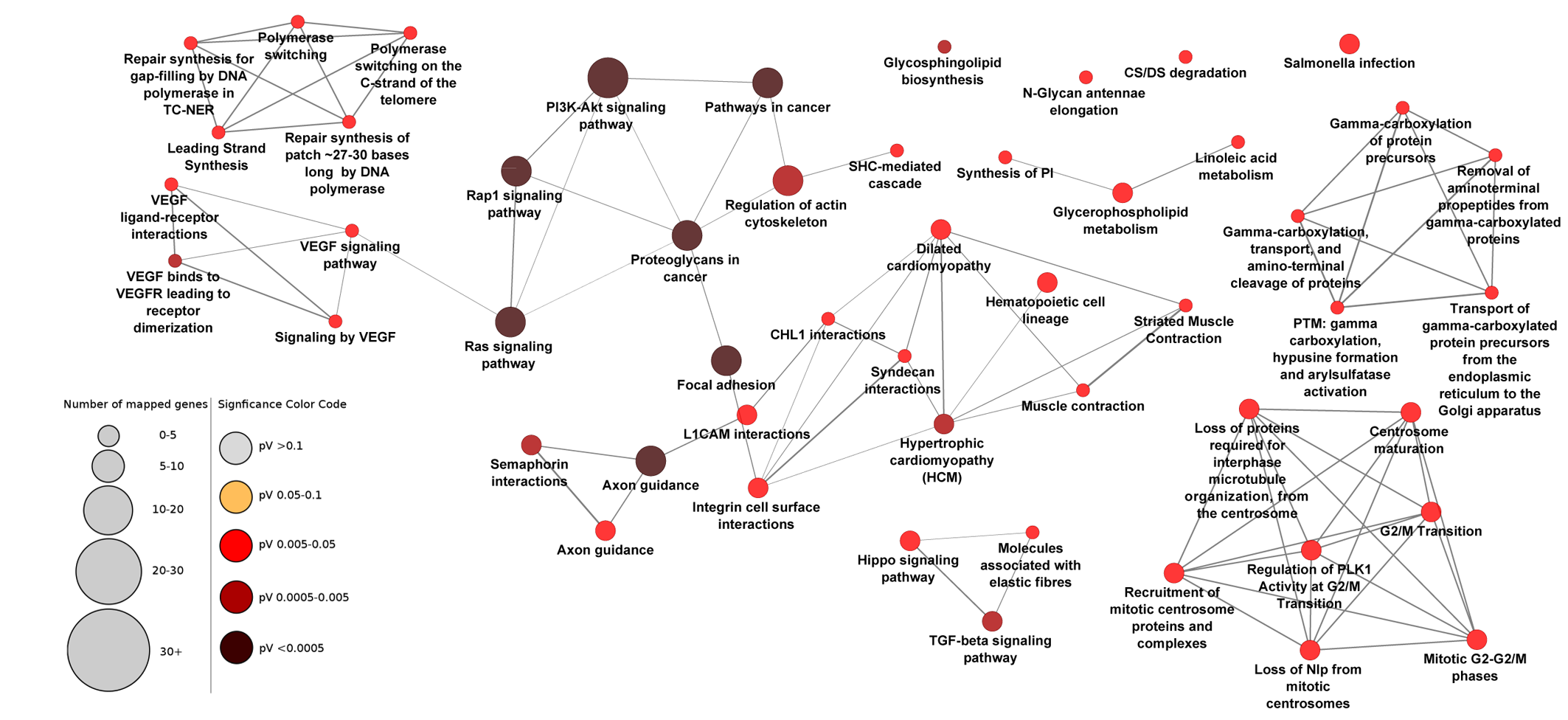

Supplement: Figure S1 — Pathway enrichment analysis with all 137 genes in the enriched PPI network revealed 51 pathways that were less connected to each other compared to pathways inferred from the central 34 genes. Pathways with adjusted P-value <0.05 are shown. [file peerj-03-1284-s002.png]
